# Muscular Response in ALS Patients during Maximal Bilateral Isometric Work of the Biceps Brachii until Fatigue

**DOI:** 10.3390/life12121978

**Published:** 2022-11-25

**Authors:** Jorge Alarcón-Jimenez, Jose Enrique de la Rubia Ortí, Julio Martín Ruiz, Nieves de Bernardo, Belén Proaño, Carlos Villarón-Casales

**Affiliations:** 1 Department of Physiotherapy, Catholic University of Valencia San Vicente Mártir, C/Quevedo, 2, 46001 Valencia, Spain; 2 Department of Nursing, Catholic University of Valencia San Vicente Mártir, C/Espartero, 7, 46007 Valencia, Spain; 3 Department of Physical Activity and Sport Sciences, Catholic University of Valencia San Vicente Mártir, C/Ramiro de Maeztu, 14, 46900 Valencia, Spain; 4 Department of Physiotherapy, European University of Valencia, Avda/Alameda, 7, 46010 Valencia, Spain

**Keywords:** muscle strength, fatigue, surface electromyography, muscular activity, exercise

## Abstract

Amyotrophic lateral sclerosis (ALS) is a neurodegenerative and fatal disease, characterized by the loss of motor neurons and progressive musculoskeletal deterioration. The clinical onset is mainly bulbar or spinal. Considering that there is no effective medical treatment, there is a need to understand the muscle activation patterns to design better physical exercise routines. The objective of this study was to determine muscle strength and fatigue in patients with ALS performing a unilateral exercise, and according to sex and type of ALS. A cross-sectional, analytical study was conducted with 23 patients. Five maximal unilateral isometric contractions were performed with the right and left biceps brachii. Muscle activation was calculated by surface electromyography bilaterally in the biceps brachii, triceps brachii, rectus femoris anterior, and tibialis anterior. The results showed more accentuated fatigue in men than in women, between the first and last contractions performed and especially on the dominant side (*p* = 0.016). In addition, there was evidence of a coactivation effect on the muscles around the work joint, which reflects a growing activation of synergists, regardless of sex or type of ALS. These findings support the use of systematic and extensive resistance exercise as a non-invasive option for maintaining the functional capacity of patients with ALS.

## 1. Introduction

Amyotrophic lateral sclerosis (ALS) is a neurodegenerative disease that causes progressive damage to motor neurons, and can lead to patient death within 16 months to 3 years from diagnosis [1]. Epidemiologically, ALS is considered to be a rare disease with an annual incidence from 1.75 to 3 cases per 100,000 people, and a prevalence of 10–12 per 100,000 in Europe, but there are significant geographical differences [2,3,4]. There is an increased risk in men (male/female ratio of 1.5), indicating that a hormonal component may be involved. However, the difference between men and women disappears with age [5,6,7]. From a clinical point of view, two main types of ALS can be identified: bulbar and spinal, depending on whether the upper or lower motor neurons, respectively, are initially affected. In both cases, a series of characteristic signs and symptoms that reflect progressive musculoskeletal deterioration can be seen. Generally, thoracic muscular dysfunction and subsequent respiratory insufficiency are the final causes of death [8]. Musculoskeletal functional impairment in ALS is associated with different alterations that decrease the functionality and quality of life of a patient, such as an increase in bone fractures due to a decrease in bone density [9,10].

At present, drugs can only provide a limited increase in survival, and symptom control is considered to be the mainstay of care for ALS patients [11]. Currently, the literature suggests that exercise could reduce deterioration in ALS patients by improving muscle strength, oxygen consumption, and fatigue [12,13,14]. Muscle strengthening and cardiovascular exercises can help to maintain functionality. Moderate aerobic training and isometric contractions produce global improvements in functional independence, which represent important benefits for patients, regardless of the type of exercise performed [15].

However, the clinical efficacy and safety of exercise in ALS patients remain uncertain due to the few published clinical studies and meta-analyses [16]. It would be necessary to analyze the real risks or benefits of exercise in detail, through controlled clinical trials and electromyographic measurements. This analysis could confirm and help to understand the benefits in terms of survival and quality of life observed in patients with ALS [17]. There is increasing evidence on the effectiveness of implementing a good exercise program, since it has been seen that it can improve fatigue, function, and quality of life of those affected by ALS [18], as well as respiratory function, mobility, and well-being in ambulatory patients [19]. Therefore, a positive causal relationship has been established between the practice of physical exercise and ALS disease [20].

Despite this, several authors agree on the need for a more comprehensive understanding of the behavior of the muscles during physical activity in patients with ALS [21], as safety and guidance for physical therapy practice remain unclear [21,22,23]. The etiology of motor fatigue is complex, involving many factors acting in multiple sites in the motor system, starting from the motor cortex with an excitatory impulse in the upper motor neurons, followed by subsequent excitation of lower motor neurons at neuromuscular junctions, sarcolemma excitability, and muscle fiber contraction [24]. Along these lines, Sanjak et al. [24] have already considered surface electromyography as a very useful tool to determine the development of the disease in terms of muscle strength and fatigue, having assessed these parameters with isometric contractions held for 5 or 30 s [25].

The main objective of this study was to determine the change in muscular activation in a series of five isometric contractions performed unilaterally with the right and left biceps brachii, in ALS patients diagnosed with bulbar or spinal symptoms. In addition, we aimed to test the level of muscular coactivation during the total time of the five contractions in each arm. This was done with five contractions maintained for 1 s with intervals of 5 s rest, to avoid generating fatigue with no acid lactic, due to the great occlusive character of a maximum 30 s contraction. Determining what occurs by fractionating the effort would help to understand the evolution of fatigue and a patient’s recovery capacity, not only in the agonist muscles already studied [26], but in the entire muscle map [27]. In addition, assessing this behavior and knowing if there is coactivation in the face of this functional demand, can open a new line of therapy, based on work that improves the functional capacity and quality of life of the ALS patient.

## 2. Materials and Methods

### 2.1. Study Design

A cross-sectional, analytical, and quantitative observational study was conducted. This trial is listed on ClinicalTrials.gov (NCT03489200), accessed on 5 April 2018.

### 2.2. Participants

Participants fulfilled the following inclusion criteria: age over 18 years; diagnosed with ALS according to the criteria of El Escorial [28] (sporadic or familial), with symptoms persisting more than 6 months; and both sexes. The clinical subtype of ALS, bulbar or spinal, was taken from the clinical records. Those females who were breastfeeding or pregnant at the time of the study were excluded. At the time of evaluation, all patients were taking Riluzole^®^ according to the standard dose prescribed by their neurologist. After applying the selection criteria, a total of 23 patients participated in the study. In order to obtain the population sample, patients were recruited thanks to the Spanish Foundation for ALS Research (FUNDELA).

### 2.3. Procedure

Once the sample was obtained, the volunteers and their families received detailed information on the study and the participants signed an informed consent form after accepting to take part in the study.

### 2.4. Measurements

To perform the surface electromyography, and with the patient in a seated position, the area designated for electrode placement was marked at the beginning of the session and wiped using cotton. The area was cleaned with alcohol and shaved with a disposable razor in order to improve electrode adherence and to avoid distortions of the electrical signal. Then, the area was cleaned again with alcohol and dried with cotton for the skin to be clean and dry. Electrode placement was longitudinal both for right and left sides:

- Biceps brachii (BB), ventral medial and external part of the humerus;
- Triceps brachii (TR), dorsal medial and internal part of the humerus;
- Anterior rectus femoris (RF), central ventral portion of the femur;
- Tibialis anterior (TA), 4 cm below the head of the fibula, longitudinal to it.

The electromyographic model used was BTS wireless FreeEmg (BTS Bioengineering). All electrodes used were Lessa Pediatric Electrode model, 30 mm diameter, and according to SENIAM [29] and Criswell [30] specifications. Two electrodes were placed with a maximum separation of 2 cm in the muscle belly, bilaterally, and the placement order by channel (Ch) number was: Ch1, right BB; Ch2, right TR; Ch3, left BB; Ch4, left TR; Ch5, right RF; Ch6, left RF; Ch7, right TA; Ch8, left TA.

The sampling frequency was 1 Khz, and each of the recordings lasted 210 s with the patient seated and at rest. After recording, the data were converted from analog to digital and saved on a hard drive for protection and later analysis in files with the extension EMT and units in millivolts. For signal analysis, a specific program, Matlab (R2021a) (Mathworks Inc., Natick, MA, USA) was used. First, a fourth order Butterworth bandpass filter was applied, between 20 and 400 Hz, to filter the signal, discarding nonspecific frequencies. Next, the rectification of the signal or RMS (root mean square) was carried out, dividing the measurement section into 100 points. The measurement unit, i.e., millivolts (mV) was converted to microvolts (uV). Subsequently, the segmentation was performed, collecting 60 s of the signal in each section corresponding to right and left biceps contraction. The smoothed average amplitude data were collected with the smoothdata function in order to be able to compare both records. Basal activation of muscles at rest was registered. Then, the patients performed five contractions of 1 s with a rest of 5 s between each contraction, according to the protocol proposed by Perry [31]. With the findpeaks function, five activation peaks were detected in both biceps, at minimum intervals of 5 s. Maximum values were also calculated with the smoothdata function. Finally, three activation zones (Zs) were established based on the temporal location of the activation peaks in order to determine the level of existing muscle coactivation. The first Z peaks between 1 and 3, the second between 3 and 4, and the last between 4 and 5.

### 2.5. Statistical Analysis

The program SPSS v.23 (IBM Corp, Armonk, NY, USA) was used for all analyses. Qualitative variables are described as proportions and quantitative variables are described as means (Ms) with standard deviations (SDs), or medians (Mds) with interquartile ranges (IQR). Normal distribution of the data was assessed with the Shapiro–Wilk test. Student’s *t*-test for independent variables (normal data) or Mann–Whitney’s U test (non-normal data) were used for mean comparisons between sex and onset categories. Student’s *t*-test for dependent variables or Wilcoxon test were used to compare right and left sides for normal and non-normal data, respectively.

A repeated measures ANOVA (RM-ANOVA) was used for comparing the 5 activation peaks and the 3 muscle Zs, for each side. Sphericity was assessed with Mauchly’s W test, and when not met, the Greenhouse–Geiser (ε) correction was applied to the degrees of freedom of the ANOVA. In all cases, the level of significance was α = 0.05. The correction of Bonferroni was used in the post hoc comparisons of the RM-ANOVA.

### 2.6. Ethical Concerns

This study was approved by the University of Valencia Institutional Review Board on Human Studies (ref. H1479983999044) and participants provided written informed consent. All the procedures followed the declaration of Helsinki [32].

## 3. Results

The sample consisted of 23 patients, with a mean age of 59 years; 56.5% of the patients were men and 43.5% of the patients were women. There were more patients with spinal ALS (65%), and the mean time from diagnosis was 24 months. The demographic and clinical characteristics of this population are shown in Table 1. When comparing the demographic and anthropometric variables between men and women, only statistically significant differences were found in weight and height, with higher means for men in both cases. A trend of younger age was seen in men (56 years), but longer diagnosis time (27 months). The BMI was 23 Kg/m^2^ for women and 25 Kg/m^2^ for men.

The comparison of the smoothed basal activation means between men and women showed a trend of greater activation in men in all the muscles analyzed, but statistically significant differences were found between sexes only in the right and left anterior rectus femoris. In addition, it was possible to see that, on the right side, the biceps presented greater activation, with a mean of 84 µV for women and 129 µV for men. In the other muscles, it was between 6 and 20 µV for women, and between 11 and 26 µV for men. For the left side, it was between 20 and 30 µV for men and between 14 and 20 µV for women.

In the smoothed activation peaks of the right and left BB, there were no statistically significant differences between women and men, although men presented somewhat higher means. In the same way, the analysis by onset type showed no statistical difference in any of the peaks, with a trend of higher activation peaks for bulbar onset patients. Complete analysis of basal data and activation peaks can be seen in Table A1.

The comparison between right and left sides for BB activation peaks showed statistically significant differences both, when comparing by gender and by ALS onset (Table 2). The right side always showed the highest activation. Regarding gender, in women, the differences between the right and left side were significant for the first three peaks, with mean differences of 113, 100, and 80 µV, respectively; in the last two (P4 and P5), the differences in activation were 66 and 61 µV. In men, the difference was statistically significant in the five peaks, with activation differences of 181, 168, 157, and 129 µV. When considering onset type, the right side was significantly higher than the left side in all peaks for spinal ALS, whereas, for bulbar ALS, the right side was significantly larger only in the first three peaks (Table 2).

**Figure 1:**
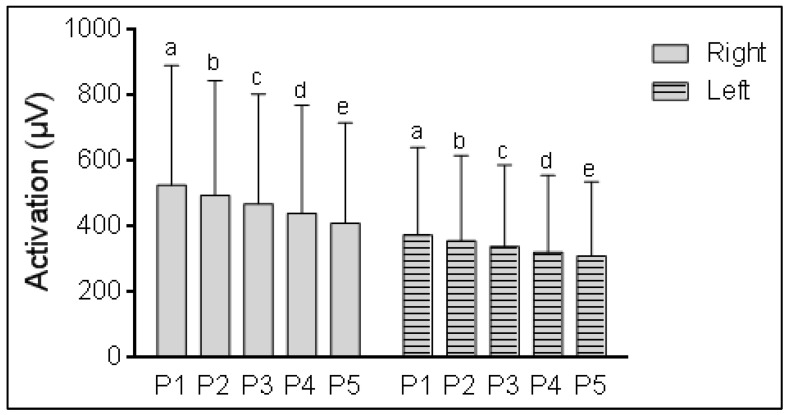
Repeated measures comparison for activation peaks (P1, P2, P3, P4, and P5) of right and left BB in ALS patients. Significance level is α = 0.05 using Bonferroni correction, statistical differences among measures are represented with letters (a, P1 ≠ Pi; b, P2 ≠ Pi; c, P3 ≠ Pi; d, P4 ≠ Pi; e, P5 ≠ Pi).

When comparing loss of strength between the peaks using RM-ANOVA, a statistically significant decrease was found between all the peaks (Figure 1), from Peak 1 to 5 for both biceps, i.e., right (df = 1.47, F = 31.9, *p* < 0.001) and left (df = 1.40, F = 26.8, *p* < 0.001). The difference between each peak was approximately 30 µV for the right side and 17 µV for the left side. Between Peaks 1 and 5, the mean differences were 115 µV and 63 µV for the right and left BB, respectively. Complete results, values of Mauchly’s W, ε, and the degrees of freedom of the Greenhouse–Geisser correction can be seen in Table A2.

**Figure 2:**
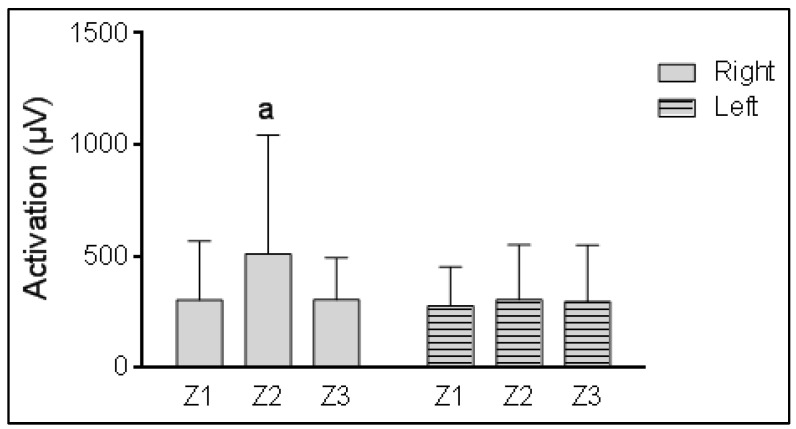
Total activation of muscular Zs (Z1, Z2, and Z3) during activity of right and left biceps brachii. Note: a, statistical difference between Z1 and Z3 (significance level is α = 0.05 using Bonferroni correction).

Regarding the activation of the work Zs (Z1, Z2, and Z3) for the right and left BB (Figure 2), statistically significant differences were found only for the right BB (df = 1.11, F = 8.22, *p* = 0.007), where Z2 showed greater activation than Z1 and Z3, reaching 500 µV; Z1 and Z3 were not statistically different, with means around 300 µV. For the left BB, there were no statistically significant differences between the work Zs (df = 1.17, F = 1.44, *p* = 0.246), always around 300 µV. In addition, no effect of sex or type of ALS diagnosis was found (Table A3).

**Figure 3:**
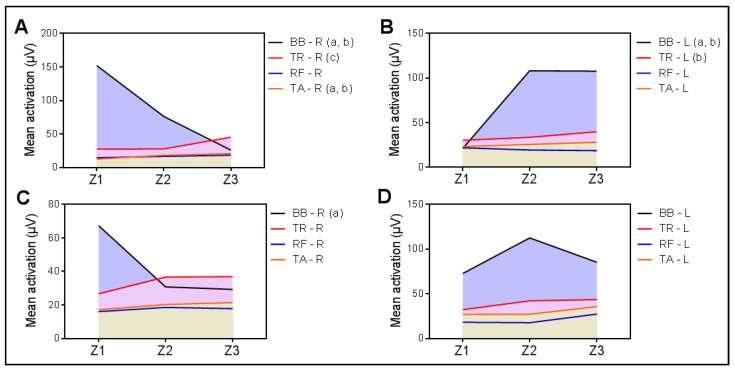
Activation of muscular Zs for each muscle during activity of biceps brachii (BB). Mean activation of the working Zs (Z1, Z2, and Z3) of BB, TR, RF, and TA muscles are represented during activity of right BB (**A**,**B**) and left BB (**C**,**D**). BB, biceps brachii; TR, triceps brachii; RF, anterior rectus femoris; TA, tibialis anterior; R, right; L, left; a, statistical differences between Z1–Z2; b, statistical differences between Z1–Z3; c, statistical differences between Z2–Z3. Significance level at α = 0.05 applying Bonferroni correction.

Finally, after comparing the activation of the work Zs for each muscle (Figure 3), it was seen that when the right BB was active, for the muscles on the right side (Figure 3A) there were statistically significant differences for the BB (df = 1.09, F = 9.74, *p* = 0.004), TR (df = 1.16, F = 6.36, *p* = 0.015), and TA (df = 1.46, F = 7.67, *p* = 0.004). The right BB activity decreased significantly from Z1 to Z2, and from Z1 to Z3. The right TR activity increased from Z2 to Z3, and the TA activity increased from Z1 to Z2, and from Z1 to Z3. For work on the left side (Figure 3B), statistically significant differences were found between the areas of the BB (df = 1.23, F = 17.02, *p* < 0.001) and the TR (df = 1.45, F = 5, *p* = 0.021). The BB activity increased significantly from Z1 to Z2, and from Z1 to Z3. The activity of the left TR also increased from Z1 to Z3. When the left BB was active, only statistically significant differences were found between the work Zs of the right BB (df = 1.43, F = 5.44, *p* = 0.016), which decreased significantly from Z1 to Z2. In the other muscles of the right side, no statistically significant differences were found (Figure 3C). Likewise, no differences were found between the Zs for the muscles of the left side (Figure 3D). The complete significance values of the RM-ANOVA during activation of both right and left BB can be seen in Table A4.

The comparison of the Z3 activation of all the muscles (RM-ANOVA) for each BB indicated statistically significant differences in both sides, when the right BB was active (df = 1.81, F = 11.53, *p* < 0.001), and when the left BB was active (df = 2.07, F = 5.82, *p* = 0.005). In the case of the right BB, it was found that the Z3 of the left BB (BB-L) was significantly greater than the rest of the muscles, except the right TR (TR-R). When the left BB was active, only Z3 activation of the right RA (RF-R) was found to be significantly lower than that of the left BB and TR (BB-L and TR-L).

## 4. Discussion

Regarding characteristic muscular alterations of ALS, the present study confirms that there is no differentiation by sex in basal activation except in one of the muscles (RA), without being agonistic in the gesture performed. Regarding muscular activation peaks for the BB, these are higher on the right side (Table 2, *p* < 0.05), which is the dominant side, as stated as well by Suzuki et al. [33]. In addition, greater fasciculations are also observed in men, increasing in conditions of fatigue, as indicated by the abrupt drops in the last activation peaks (181-168-168-157-129 µV) in this sex as compared with the more attenuated decrease in women (113-100-80-66-61 µV). This could also be due to higher peak values in the right side of male patients, although not statistically different from females (Table A1).

This marked laterality in the ability to generate muscle tension also exists depending on the type of ALS. In spinal ALS, there are significant differences in the five activation peaks, with greater imbalance between the right and left biceps (Table 2, *p* < 0.05). In bulbar ALS, greater fatigue and loss of activation is detected in the first three peaks (Table 2, *p* = 0.1), with no difference in the other two peaks, where a smaller mean difference around 70 µV can be seen. In spinal onset patients, the differences are around 100 µV for all the peaks, which implies lower activation, i.e., below 300 µV, in P5 for this group. This confirms that this variant is more related to fatigue and to more components of physical condition, mainly limb musculature, as has already been indicated by Sznajder et al. [18]. On the contrary, bulbar ALS patients have greater difficulties in cardiorespiratory musculature. In addition, the trend favoring better limb performance in men, has also been suggested before, together with an effect of onset type, the male spinal ALS patients were healthier (reaching normotension) than women, especially with bulbar ALS [34]. The increased tensions produced in an isometric contraction is effective for rapid strength gains and the generation of a greater metabolic load [35].

Increasing activation is fundamental in muscle tissue efficiency, as has been indicated by Pallarés et al. [36], and this type of isometric conditioning has been recommended in several neuropathies, combined with sensorimotor practices [37].

After the application of this study and with the muscular behavior observed in the five isometric peaks, it could be assumed that dividing the work in short intervals and alternating contraction and recovery favors the fatigue to be more attenuated than what has been observed so far [25], and may allow longer-lasting efforts by causing less peripheral fatigue.

Although it is not yet possible to conclusively generalize how to improve the response to this fatigue in ALS patients [38], it has been shown that muscle pathology is attributed to neuromuscular degeneration and not to intrinsic defects of the muscle fibers. However, the surviving muscle fibers maintain adaptive capacity to exercise, using muscle compensations, which delay the evolution of the disease [39]. This is particularly relevant, since coactivation allows a patient to continue doing moderate muscular work with greater motor stability [40].

This study shows that there is a pattern of muscle coactivation with higher activity in Z2 during right BB activity (Figure 2), regardless of gender and onset type (Table A3). When each muscle coactivation is analyzed (Figure 3), a pattern involving two of the three work Zs (Z1 and Z3) can be seen with the active right biceps (*p* = 0.01) and with the left (*p* = 0.005), with a decrease in Z activity of the BB and an increase in Z activity of the TR across time, for the right side, indistinctively of the active BB, right or left. The coactivation of left side muscles has a trend to increase from Z1 to Z3, indistinctively of the active BB, which is significant for Z peaks of the BB and TR. The other muscles, FR and TA, remain almost constant in both scenarios. This model shows that there is a global synergistic work of the muscles, regardless of whether or not the agonist muscle performs an action of analytical nature. This finding coincides with Mausehund et al. [26] who reported that, in activities close to muscle failure, there was an evident coactivation, especially in unilateral exercises. The behaviors of the biceps and triceps brachii motor units are quite similar and progressive as the load progresses and contraction is maximized [41,42]. It is of great relevance, even for postural stability, that the nervous system recalibrates and compensates the system with agonists that help to maintain stability [43] even in actions with risk of injury, where the muscles work to avoid joint damage [44].

The results show that the individuals present significant differences in the muscle activation rhythm (Z1 and Z3, but not Z2), in accordance with those individuals without pathologies in a progressive aerobic cyclic test (ergospirometry), especially in the first work Z (without CO_2_ production), without showing differences in the second and third, with greater metabolic instability [45]. The data of the present study, therefore, reaffirm the fact that maintaining greater coactivation in sedentary adults between 55 and 65 years of age is very important, although it is conditioned by the individual characteristics of each patient and generalizing benefits is difficult [27], and that it has a greater effect in those patients with spinal involvement [21].

It has been shown that exercise can significantly improve functional capacity and lung function in ALS patients [16]. An adequate load and density are key for the design of a correct treatment, since in some pathologies it has been found that excessive coactivation is associated with a higher level of spasticity [46], and it should be the plan to follow in order to standardize exercise protocols.

The initial guideline to follow would focus on practicing exercise twice a week (no changes have been reported in either of the functions or in fatigue with a greater number of sessions [47]), in concurrent 12-week programs, since it has been shown that focusing attention on a single component of physical condition was ineffective [15,20,23], showing that multi-component programs would be the recommended line of work [48].

However, some limitations need to be highlighted. Firstly, the small sample size invites us to interpret the findings with caution, without being able to generalize them by sex or type of ALS. Secondly, having performed an analytical and uniarticular exercise, the comparison with another of a more global nature is pending; this experiment could reveal similarities and differences depending on the type of contraction performed. Finally, in future studies, different contraction and recovery times should be used to calibrate the most suitable ratio between both in ALS patients, making it possible to establish minimum recovery times for optimal activation during exercise.

## 5. Conclusions

Therefore, the conclusions of the study are that regardless of the type of ALS or sex of the patient, when fatigue increases in a high-density maximum force exercise on the agonist musculature, there is a synergistic neuronal response from the antagonistic muscles and from the muscle groups far from the joint responsible for the contraction. These conclusions seem to indicate that the design of specific programs, including isometric contractions, which affect this adaptive capacity could represent one of the most accessible non-invasive options for maintaining functional capacity and improving quality of life in ALS patients.

## Figures and Tables

**Table 1:** Demographic and clinical data of the study sample.

Sex	N	%
Male	13	56.5
Female	10	43.5
**Onset type**	N	%
Bulbar (m)	8 (5)	34.8
Spinal (m)	15 (8)	65.2
	M ± SD	Md (IQR)
Age (years)	59 ± 10.53	58 (14.5)
Disease duration (mos)	24 ± 17.74	18 (23)
Weight (Kg)	68 ± 9.47	71 (15.4)
Height (cm)	166 ± 7.47	166 (13.5)
BMI (Kg/m^2^)	25 ± 2.71	25 (4.2)

M, mean; SD, standard deviation; Md, median; IQR, interquartile range; m, males; mos, months.

**Table 2:** Mean of the differences between the right and left sides for activation peaks (P1–P5) of the biceps brachii, according to sex and ALS onset type.

Female	MD	SD	t/w	t/z	df	*p*-Value
P1 R-L	113.7	142.7	t	2.52	9	0.033
P2 R-L	100.6	129.7	w	−2.09	9	0.037
P3 R-L	80.1	116.6	w	−1.99	9	0.047
P4 R-L	66.3	117.9	w	−1.38	9	0.169
P5 R-L	61.5	106.8	w	−1.38	9	0.169
**Male**	**MD**	**SD**	**t/w**	**t/z**	**df**	***p*-Value**
P1 R-L	181.0	231.6	t	2.82	12	0.016
P2 R-L	168.0	215.2	t	2.82	12	0.016
P3 R-L	168.9	205.4	t	2.96	12	0.012
P4 R-L	157.5	210.7	t	2.70	12	0.019
P5 R-L	129.1	185.9	t	2.50	12	0.028
**Spinal onset**	**MD**	**SD**	**t/w**	**t**	**df**	***p*-Value**
P1 R-L	169.0	232.3	t	2.8	14	0.014
P2 R-L	151.7	213.4	t	2.8	14	0.016
P3 R-L	147.0	207.3	t	2.7	14	0.016
P4 R-L	136.4	203.4	t	2.6	14	0.021
P5 R-L	113.6	178.0	t	2.5	14	0.027
**Bulbar onset**	**MD**	**SD**	**t/w**	**t**	**df**	***p*-Value**
P1 R-L	119.3	109.6	t	3.1	7	0.018
P2 R-L	114.4	112.1	t	2.9	7	0.023
P3 R-L	99.0	92.2	t	3.0	7	0.019
P4 R-L	83.2	125.6	t	1.9	7	0.103
P5 R-L	73.7	114.2	t	1.8	7	0.111

MD, mean of the differences; SD, standard deviation; R, right; L, left; t, normal distribution; w, non-normal distribution; df, degrees of freedom.

## Data Availability

This trial is listed on ClinicalTrials.gov (NCT03489200), accessed on 5 April 2018.

## Appendix A

**Table A1:** Full results of basal activation of upper and lower limb muscles from men and women with ALS. Activation peaks of the biceps brachii according to gender and onset type.

Basal Activation (by Gender)	Female		Male					
	M	SD	M	SD	t/u	t/z	df	*p*-Value
BB-R (µV)	83.8	64.2	129.2	76.7	t	−1.51	21	0.146
BB-L (µV)	19.8	8.2	30.0	20.2	u	−1.18	21	0.239
TR-R (µV)	19.0	6.1	25.9	14.5	u	−0.92	20	0.356
TR-L (µV)	18.6	10.8	29.8	17.1	u	−1.45	20	0.147
RA-R (µV)	5.8	2.0	16.6	9.5	t	−3.96	13.4	0.002
RA-L (µV)	11.6	5.5	26.7	16.2	t	−3.12	15.4	0.007
TA-R (µV)	8.0	5.3	10.9	6.7	u	−0.73	20	0.468
TA-L (µV)	14.7	8.8	20.3	17.5	u	−0.46	20	0.644
**Peak activation (by gender)**	**Female**		**Male**					
	**M**	**SD**	**M**	**SD**	**t/u**	**t/z**	**df**	***p*-Value**
P1-R	417.5	353.0	609.8	365.7	t	−1.27	21	0.218
P2-R	389.7	337.3	576.7	349.9	t	−1.29	21	0.211
P3-R	357.2	311.3	555.9	338.7	t	−1.44	21	0.164
P4-R	327.5	300.4	526.8	337.4	t	−1.47	21	0.156
P5-R	310.7	283.0	487.0	310.5	t	−1.40	21	0.176
P1-L	303.8	282.8	428.8	251.6	t	−1.12	21	0.275
P2-L	289.1	280.1	408.6	243.2	u	−1.30	21	0.193
P3-L	277.1	268.8	387.0	231.2	u	−1.36	21	0.172
P4-L	261.1	260.9	369.3	209.7	u	−1.43	21	0.154
P5-L	249.2	247.0	357.9	205.8	u	−1.43	21	0.154
**Peak activation (by onset type)**	**Spinal**		**Bulbar**					
	**M**	**SD**	**M**	**SD**	**t/u**	**t**	**df**	***p*-value**
P1-R	501.0	414.1	573.3	269.1	t	−0.51	19.9	0.619
P2-R	471.6	397.6	539.9	254.6	t	−0.50	20.1	0.623
P3-R	451.3	377.9	503.6	255.9	t	−0.35	21	0.730
P4-R	434.6	367.4	450.6	270.5	t	−0.11	21	0.915
P5-R	401.2	335.6	427.6	259.9	t	−0.19	21	0.849
P1-L	332.0	266.6	454.0	265.7	t	−1.05	21	0.307
P2-L	319.9	265.6	425.5	254.0	t	−0.92	21	0.368
P3-L	304.3	254.4	404.6	239.4	t	−0.92	21	0.369
P4-L	298.2	250.4	367.3	208.3	t	−0.67	21	0.513
P5-L	287.6	239.0	353.9	207.8	t	−0.66	21	0.516

BB, biceps brachii; TR, triceps bracii; RA, anterior rectus femoris; TA, tibialis anterior; P, peaks of activation of the BB; M, mean; SD, standard deviation; R, right; L, left; t, normal distribution; u, non-normal distribution; df, degrees of freedom.

**Table A2:** RM-ANOVA for comparison of the 5 activation peaks of the right and left BB.

	M	SD	N	Mauchly’s W				ANOVA-Greenhouse–Geiser Correction				
				W	X^2^	gl	Sig.	ε	df	CM	F	*p*-Value
P1-R	526.2	365.3	23	0.023	76.9	9	<0.001	0.368	1.47	128,428.3	31.9	<0.001
P2-R	495.4	349.7	23									
P3-R	469.5	335.2	23									
P4-R	440.2	330.5	23									
P5-R	410.4	305.5	23									
	**M**	**SD**	**N**	**W**	**X^2^**	**gl**	**Sig.**	**ε**	**df**	**CM**	**F**	***p*** **-Value**
P1-L	374.4	266.9	23	0.016	83.4	9	<0.001	0.350	1.40	43,372.0	26.8	<0.001
P2-L	356.6	260.8	23									
P3-L	339.2	248.6	23									
P4-L	322.3	234.2	23									
P5-L	310.7	226.1	23									

P, peak; M, mean; SD, standard deviation; W, W de Mauchly; df, degrees of freedom; ε, for Greenhouse–Geiser correction; CM, cuadratic mean.

**Table A3:** Activation of the 3 working areas (Zs) during BB activity, analysis by gender and ALS onset type.

Gender	Female		Male	
BB-R	M	SD	M	SD
Z1	186.3	91.6	395.0	317.4
Z2	319.6	222.7	658.5	654.3
Z3	216.5	151.1	374.0	191.0
**BB-L**	**M**	**SD**	**M**	**SD**
Z1	186.5	102.7	347.6	190.5
Z2	213.7	156.4	377.1	282.7
Z3	210.3	140.7	364.4	303.3
**Onset type**	**Spinal**		**Bulbar**	
**BB-R**	**M**	**SD**	**M**	**SD**
Z1	314.2	323.2	285.5	95.8
Z2	542.8	641.1	451.8	249.6
Z3	305.7	217.3	305.3	132.1
**BB-L**	**M**	**SD**	**M**	**SD**
Z1	278.9	204.3	275.0	114.9
Z2	310.5	286.0	297.6	162.3
Z3	307.4	296.2	278.7	161.8

R, right; L, left; M, mean; SD, standard deviation. No significant differences were found (α = 0.05).

**Table A4:** RM-ANOVA for comparison of Zs for each muscle during activation of the right and left BB.

Right BB									
Muscle	M	SD	N	RM-ANOVA	Muscle	M	SD	N	RM-ANOVA
BB-R Z1	152.3	189.5	23	gl = 1.096; F = 9.737; *p* = 0.004	BB-L Z1	21.2	17.1	23	gl = 1.239; F = 17.019; *p* < 0.001
BB-R Z2	76.4	160.1	23		BB-L Z2	108.1	92.4	23	
BB-R Z3	26.0	15.9	23		BB-L Z3	107.9	104.5	23	
TR-R Z1	27.5	19.8	23	gl = 1.165; F = 6.361; *p* = 0.015	TR-L Z1	30.3	31.8	23	gl = 1.450; F = 5.002; *p* = 0.021
TR-R Z2	27.9	27.0	23		TR-L Z2	33.7	28.7	23	
TR-R Z3	45.5	40.4	23		TR-L Z3	40.0	37.8	23	
RA-R Z1	14.8	16.5	23	gl = 1.500; F = 2.620; *p* = 0.101	RA-L Z1	22.0	18.0	23	gl = 2; F = 2.877; *p* = 0.067
RA-R Z2	16.8	18.9	23		RA-L Z2	19.4	16.8	23	
RA-R Z3	18.5	20.5	23		RA-L Z3	18.6	13.7	23	
TA-R Z1	12.8	20.2	23	gl = 1.461; F = 7.666; *p* = 0.004	TA-L Z1	23.3	27.4	23	gl = 2; F = 1.956; *p* = 0.154
TA-R Z2	18.5	24.0	23		TA-L Z2	25.6	26.9	23	
TA-R Z3	20.9	31.5	23		TA-L Z3	28.1	32.5	23	
**Left BB**									
**Muscle**	**M**	**SD**	**N**	**RM-ANOVA**	**Muscle**	**M**	**SD**	**N**	**RM-ANOVA**
BB-R Z1	67.3	72.2	23	gl = 1.434; F = 5.442; *p* = 0.016	BB-L Z1	72.9	82.4	23	gl = 1.432; F = 2.613; *p* = 0.104
BB-R Z2	30.8	42.2	23		BB-L Z2	112.6	105.7	23	
BB-R Z3	29.3	24.5	23		BB-L Z3	85.4	100.4	23	
TR-R Z1	26.7	23.3	23	gl = 1.575; F = 0.890; *p* = 0.397	TR-L Z1	32.3	29.7	23	gl = 2; F = 4.177; *p* = 0.022
TR-R Z2	36.7	43.3	23		TR-L Z2	42.2	38.9	23	
TR-R Z3	36.9	37.7	23		TR-L Z3	43.6	38.9	23	
RA-R Z1	16.0	18.6	23	gl = 1.386; F = 1.115; *p* = 0.321	RA-L Z1	18.2	12.4	23	gl = 1.012; F = 1.045; *p* = 0.319
RA-R Z2	18.6	21.1	23		RA-L Z2	17.6	13.7	23	
RA-R Z3	17.8	18.1	23		RA-L Z3	27.3	51.3	23	
TA-R Z1	17.1	22.7	23	gl = 1.256; F = 1.787; *p* = 0.193	TA-L Z1	27.0	34.9	23	gl = 1.134; F = 2.574; *p* = 0.118
TA-R Z2	20.3	30.7	23		TA-L Z2	27.2	35.5	23	
TA-R Z3	21.4	28.7	23		TA-L Z3	35.6	55.4	23	

BB, biceps brachii; TR, triceps brachii; RA, anterior rectus femoris; TA, tibialis anterior; R, right; L, left; M, mean; SD, standard deviation; RM-ANOVA, repeated measures ANOVA.
